# Retraction: Ligustrazine inhibits the proliferation and migration of ovarian cancer cells via regulating miR-211

**DOI:** 10.1042/BSR-2020-0199_RET

**Published:** 2024-10-07

**Keywords:** EMT, LSZ, miR-211, SK-OV-3

This article “Ligustrazine inhibits the proliferation and migration of ovarian cancer cells via regulating miR-211” DOI: 10.1042/BSR20200199 is being retracted from *Bioscience Reports* at the request of the Editor-in-Chief and the Editorial Board. This follows receipt of a notification from a reader, alerting the Editorial Board to two images in this paper that also appear in separate articles by different authors. Specifically:

- Figure 2B OVCAR-3 2 mM LSZ panel which seems to appear in Figure 2D SW620 sh-nc panel from Wang et al, 2022 (DOI: 10.1186/s12967-022-03240-x)
- Figure 5B Blank control panel which seems to appear in Figure 2D Migration Control panel from Xiao et al, 2022 (DOI: 10.1080/21655979.2022.2066760).

The authors contacted the Journal requesting a correction to Figure 2B and Figure 5B, after which the Editorial Office informed the authors of the concerns raised. The authors responded to the Journal's queries and provided data for Figure 2B and Figure 5B, for which no further issues were identified. The authors originally stated there were mistakes saving and using the data for Figure 2B and Figure 5B, then later stated these data were correct. Given the images also appear in other publications with a wider field of view, the Journal has concerns surrounding the storage, handling, and scientific validity of the data in this paper. The authors do not agree to the retraction. The Editorial Board stands by the decision to retract.

